# FIVES: A Fundus Image Dataset for Artificial Intelligence based Vessel Segmentation

**DOI:** 10.1038/s41597-022-01564-3

**Published:** 2022-08-04

**Authors:** Kai Jin, Xingru Huang, Jingxing Zhou, Yunxiang Li, Yan Yan, Yibao Sun, Qianni Zhang, Yaqi Wang, Juan Ye

**Affiliations:** 1 Department of Ophthalmology, the Second Affiliated Hospital of Zhejiang University, College of Medicine, Zhejiang University, Hangzhou, 310009 China; 2 School of Electronic Engineering and Computer Science, Queen Mary University of London, London, E1 4NS United Kingdom; 3 College of Computer Science and Technology, Hangzhou Dianzi University, Hangzhou, 310018 China; 4 College of Media Engineering, Communication University of Zhejiang, Hangzhou, 310018 China

**Keywords:** Image processing, Databases

## Abstract

Retinal vasculature provides an opportunity for direct observation of vessel morphology, which is linked to multiple clinical conditions. However, objective and quantitative interpretation of the retinal vasculature relies on precise vessel segmentation, which is time consuming and labor intensive. Artificial intelligence (AI) has demonstrated great promise in retinal vessel segmentation. The development and evaluation of AI-based models require large numbers of annotated retinal images. However, the public datasets that are usable for this task are scarce. In this paper, we collected a color fundus image vessel segmentation (FIVES) dataset. The FIVES dataset consists of 800 high-resolution multi-disease color fundus photographs with pixelwise manual annotation. The annotation process was standardized through crowdsourcing among medical experts. The quality of each image was also evaluated. To the best of our knowledge, this is the largest retinal vessel segmentation dataset for which we believe this work will be beneficial to the further development of retinal vessel segmentation.

## Abstract (summary)

**Table:** 

Measurement(s)	retinal image segmentation
Technology Type(s)	manual segmentation
Sample Characteristic - Organism	Homo sapiens
Sample Characteristic - Environment	hospital
Sample Characteristic - Location	Zhejiang Province, China

## Background & Summary

**Fig. 1:**
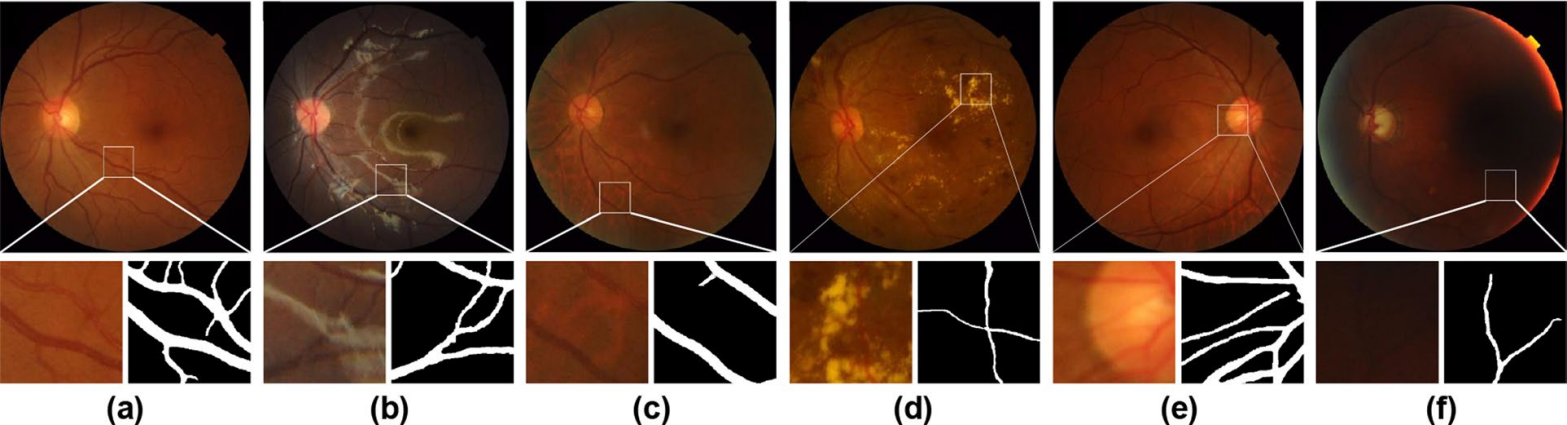
Illustration of fundus photographs and the challenges of retinal vessel segmentation: (**a**) A normal high-quality fundus image, with the corresponding segmentation result shown in a detailed view. (**b**) A child’s fundus image showing the retinal nerve fibers blocking the view of the connections between vessels. (**c**) An elderly person’s fundus image showing a tessellated retina, which can be misrecognized as vessels. (**d**) A fundus image showing pathological changes related to diabetic retinopathy. (**e**) A fundus image with poor focus. (**f**) A fundus image with insufficient exposure.

Retinal vasculature is a reliable reflection of vessel morphologic changes, which have been demonstrated to relate to numerous clinical conditions, including both ophthalmological and systemic diseases. Diabetic retinopathy (DR), age-related macular degeneration (AMD), and glaucoma are major blindness-causing diseases^1^. Multiple studies have investigated the relationship between retinal vasculature characteristics and these ocular diseases^2–4^. Some life-threatening systemic diseases can also be reflected in fundus images, such as cardiovascular diseases^5^ and neurological disorders^6^. Several retinal vessel morphometrics are thought to be linked to disease risk and progression^7–10^. Retinal vessel segmentation, which means the extraction of visible vasculature from a fundus image (Fig. 1a), is the preliminary step to objectively assessing the fundus image vasculature and quantitatively interpreting the morphometrics.

Retinal vessel segmentation is a challenging task, considering that age, ophthalmic disease, and photograph conditions all have a considerable impact on segmentation. In children, there is a prominent sheen from the retinal nerve fibers near the vessels and macula, blocking the view of the connections between vessels (Fig. 1b). In elderly adults, hypoplasia of the retinal pigment epithelium leaves the choroidal vessels visible, similar to the retinal vasculature (Fig. 1c). Microaneurysms, hemorrhages, and exudates can be seen on fundus images of diabetic retinopathy patients (Fig. 1d). Eye movements and improper operations can result in loss of focus (Fig. 1e) and insufficient exposure (Fig. 1f).

In retinal image analysis, manual segmentation of retinal blood vessels is extremely time consuming and labour intensive, unlike other retinal image preprocessing tasks such as artery-vein classification, which is important but can be done manually while conducting medical studies. Automated and semiautomated vessel segmentation is thus an indispensable process for efficient quantitative interpretation of the retinal vasculature. Many studies have been conducted on retinal vessel segmentation, including conventional algorithms^11^ and artificial intelligence (AI) based segmentation^12^. AI based methods have been intensively studied in recent years. The development and evaluation of AI-based methods requires datasets with large amounts of annotated images.

Several retinal vessel segmentation datasets, which are summarized in Table 1, have been established for public use: STARE^13^, DRIVE^14^, ARIA^15^, REVIEW^16^, CHASEDB1^17^, HRF^18^, etc. Nearly all retinal vessel segmentation work has been carried out on these datasets, including state-of-the-art vessel segmentation algorithms, such as SCS-Net^19^, the NFN + model^20^ and the MS-DRIS-GP model^21^. There are also algorithms that can do a simultaneous segmentation and artery-vein classification developed on these datasets, such as the work of Hemelings *et al*.^22^.

**Table 1:** Summarization of publicly available retinal vessel segmentation datasets.

Dataset		Year	Number	Resolution	Disease	Annotators
STARE		2000	20	605 × 700	10 healthy, 10 diseases	2
DRIVE		2004	40	768 × 584	33 healthy, 7 DR	3
ARIA		2006	161	576 × 768	61 healthy, 59 DR, 23 AMD	2
REVIEW	HRIS	2008	4	3584 × 2438	16 DR	3
	VDIS		8	1360 × 1024		
	CLRIS		2	2160 × 1440		
	KPIS		2	288 × 119, 170 × 92		
CHASEDB1		2011	28	990 × 960	28 healthy	2
**FIVES (proposed)**		**2021**	**800**	**2048 × 2048**	**200 healthy, 200 AMD, 200 DR, 200 glaucoma**	**Group**

**Fig. 2:**
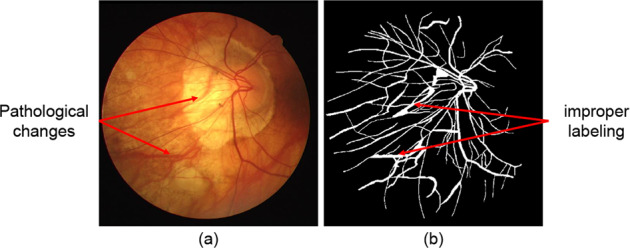
Example of mislabeling from public dataset: (**a**) the primary fundus photograph from the DRIVE dataset with red arrow referring to strip-like pathological changes, (**b**) the ground truth provided in the dataset with red arrow referring to the improper labeling of strip-like pathological changes as retinal vessels.
- Third, image quality, which is an important factor affecting the segmentation performance, is not evaluated. Providing the evaluation of images will be useful for further in-depth research into the question of how the performance of methods is affected by image quality.

However, there are some inherent drawbacks of these popular datasets:

- First, the images included in these datasets are small in quantity and imbalanced in disease distribution, and most of them are of low resolution. A small quantity may lead to overfitting when using deep learning. The distribution of disease is rather imbalanced, for which disease-specific analysis cannot be conducted. Some of the most commonly used datasets have resolutions of less than 1000 × 1000 pixels^23^.
- Second, the annotation process has not been standardized by healthcare professionals, so inappropriate annotations may occur. For example, some pathological changes will form a strip-like structure and should not be labelled as retinal vessels, as is shown in Fig. 2.

Taking all of these factors into consideration, it would be meaningful to establish a public retinal vessel segmentation dataset with pixelwise annotation.

In this paper, we propose a fundus image vessel segmentation (FIVES) dataset consisting of 800 high-resolution color fundus photographs with pixelwise manual annotation through standard crowdsourcing among medical staff. For each image, 3 labels were provided: disease, pixelwise vessel annotation and image quality scores. Our major contributions can be summarized as follows:

- **Image**: The dataset contains 800 high-resolution images of normal eyes and 3 different eye diseases, with 200 images in each category.
- **Annotation**: Pixelwise annotation was performed by a group of trained medical staff and verified by experienced ophthalmologists who had been annotating for hundreds of hours. The annotation group was made up of 3 ophthalmic practitioners as senior annotators and 24 medical staff who were knowledgeable regarding retinal anatomy as junior annotators.
- **Evaluation**: The quality of images was evaluated from three perspectives using an automatic algorithm and further corrected by retinal specialists.

We believe that the publication of the FIVES dataset will considerably facilitate computer-aided retinal vessel segmentation research and promote translation from technology to clinical use.

## Methods

### Data collection

**Fig. 3:**
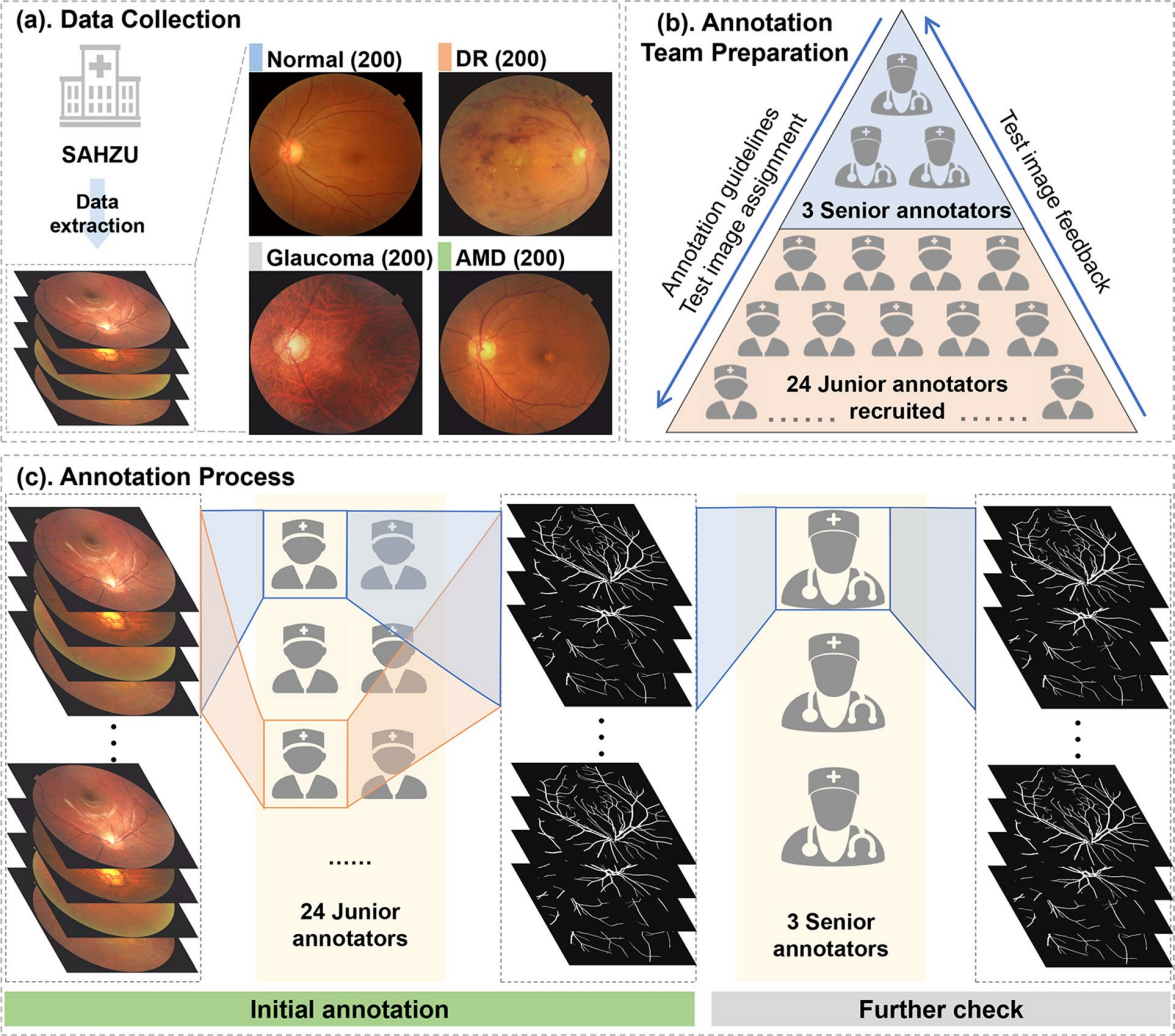
Workflow of the establishment of the proposed dataset. (**a**) Eight hundred color fundus photographs were collected in the Ophthalmology Center at the Second Affiliated Hospital of Zhejiang University (SHAZU). These photographs comprised 200 images from patients diagnosed with diabetic retinopathy (DR), 200 from patients with glaucoma, 200 from patients with age-related macular degeneration (AMD), and 200 from normal controls. (**b**) The annotation team was made up of 3 senior annotators and 24 junior annotators who had completed guideline-based training and image annotation tests. (**c**) The interface of the specifically designed annotating software used in this study. (**d**) The annotation process consisted of initial annotation by junior annotators and further verification by senior annotators. Each image was randomly assigned to 2 annotators, and the common pixels annotated by both of them were included as the result of initial annotation.

Eight hundred color fundus photographs were collected from 2016 to 2021 in the Ophthalmology Centre at the Second Affiliated Hospital of Zhejiang University (SHAZU) (Fig. 3a). These images are from 573 patients, with ages ranging from 4 to 83 years. Written informed consent complying with the requirement of the Medical Ethics Committee of SHAZU was signed by every participant when it was decided that their photographs would be adopted by scientific research. The study followed the tenets of the Helsinki Declaration and was approved by the Medical Ethics Committee of SAHZU. The study has been registered on ClinicalTrials.gov with trial registration number NCT04718532.

Patients underwent comprehensive systemic and ocular examinations to establish a diagnosis. Before photographing, 2 to 3 drops of 1.0% tropicamide phenylephrine were used for pupil dilation of every examined eye. Pupils with diameters over 7 millimeters were considered fully dilated, and fundus photographs were taken. The images were captured by tabletop TRC-NW8 fundus cameras at a 50° field of view (Topcon Medical Systems, Tokyo, Japan) and centered at the macula. For a 50° field of view, both the macula and the optic disc could be seen in the image. The photographing process was conducted by experienced examiners. For each examined eye, only one photograph was included in the final dataset. For each patient, both eyes were examined. All the images were collected and manually selected to form the final dataset. The manual selection considered both disease diagnosis and image readability. Selection criteria considering disease diagnosis can be found below, and we have intentionally included about 5% images with poor readability assessed by experienced ophthalmic doctors to reflect real clinical situation. Pictures were saved in PNG format with a resolution of 2048 × 2048 pixels. No image compression was conducted during the image capturing, annotation and dataset uploading processes.

### Disease diagnosis

In this study, only patients with a clearly ascertained diagnosis were included. The diagnostic procedure started with first-contact doctors and was further reviewed by experienced ophthalmologic specialists. The diagnosis criteria complied with most updated American Academy of Ophthalmology’s guidelines at the time when the picture was taken. Details are described below:

For DR, both type 1 and type 2 diabetes mellitus (DM) patients were considered. The diagnosis of DM was based on the criteria proposed by the American Diabetes Association^24^. Examinations necessary for the diagnosis of DM were conducted before image capture, including slit-lamp examination, ultrawide fundus photography, optical coherence tomography and fundus florescence angiography, when necessary. Images with laser scars were also included to mimic real clinical practice.

Patients with age-related macular degeneration (AMD) underwent thorough ocular examinations, including slit-lamp examination, fundus photography, optical coherence tomography (OCT) and fundus fluorescein angiography, if necessary. Pathological features in fundus images include drusen, exudate, geographic atrophy or hemorrhage. The diagnostic procedure was conducted according to the AAO’s Preferred Practice Pattern for AMD^25^.

For glaucoma, both open-angle glaucoma and angle-closure glaucoma were included in this study. Patients of this kind often present headache, decreased vision, elevated intraocular pressure (IOP) and optic nerve damage, which can be seen in fundus photographs. The diagnosis of glaucoma is based on clinical complaints, IOP, fundus photography, visual field examination and optical coherence tomography. The detailed diagnostic criteria can be found in AAO’s Preferred Practice Pattern of glaucoma^26,27^.

The exclusion criteria included patients with an uncertain diagnosis, patients with multiple diseases, patients with excessive opacity in refractive media, patients with systemic diseases other than DM that can affect the retina, patients with familial ocular diseases, and patients with ocular trauma.

### Vessel annotation

The annotation process started with the recruitment of annotators (Fig. 3b). The recruitment message was posted online and shared via social media, requesting that medical workers who were willing to participate and well aware of the retina anatomy could join in this work. Finally, the annotation group consisted of 3 ophthalmic practitioners with clinical ophthalmic experience and 24 medical staff meeting the recruitment criteria.

The training of annotators was an important part of the annotation process (Fig. 3b). A detailed annotation guideline was made by 3 ophthalmic practitioners of SAHZU. For each annotator, having learned the annotation guidelines, 5 test images, including 1 normal, 2 DR, 1glaucoma and 1 AMD, were assigned and retrieved after their initial annotation. The test images were evaluated by the ophthalmic practitioner one by one. If the annotation result was not satisfiable, the image was sent back to the student for relabeling. If the results of relabeling were still unacceptable, the annotators could not enter the next stage of annotation work. If the test images were thought to be appropriately labeled, more images would be assigned. Annotators were paid differently according to their annotation performance, which was qualitatively evaluated by senior annotators and project managers based on three aspects: false positive pixels, false negative pixels and overall vasculature.

The annotation was achieved using specifically designed annotating software. Figure 3c shows the annotating interface. Annotators were asked to use the pencil tool to color the pixel white if they thought it was a vessel pixel based on the appearance. In some images, the vessels seemed broken due to various reasons. To ensure that the annotated pixels were from vessels and reduce false positive annotation, annotators were told that no modification should be made just to maintain the continuity of the vasculature. The final vessel segmentation ground truth is a two-color image, with white pixels meaning vessel and black for non-vessel pixels. The resolution of the ground truth is 2048 × 2048 pixels, coordinated with the original fundus image.

In the official annotation stage (Fig. 3d), each annotator was assigned over 30 images. Each image was annotated by 2 annotators. The pixels annotated by the 2 annotators in common were included as the final ground truth of the specific image to reduce the number of false positive pixels. Annotating one image would take approximately 3–5 hours. The overall annotation process began in December 2020 and ended in June 2021. After being annotated by junior annotators, the images were further reviewed by senior annotators to correct errors, such as misidentification of the choroidal vessels as retinal vessels. If there were clear mistakes, the senior annotators would directly correct them when reviewing. If the senior annotators disagreed with the annotation results but were not certain, they would discuss the matter amongst themselves and then make the final decision.

### Image quality assessment

**Fig. 4:**
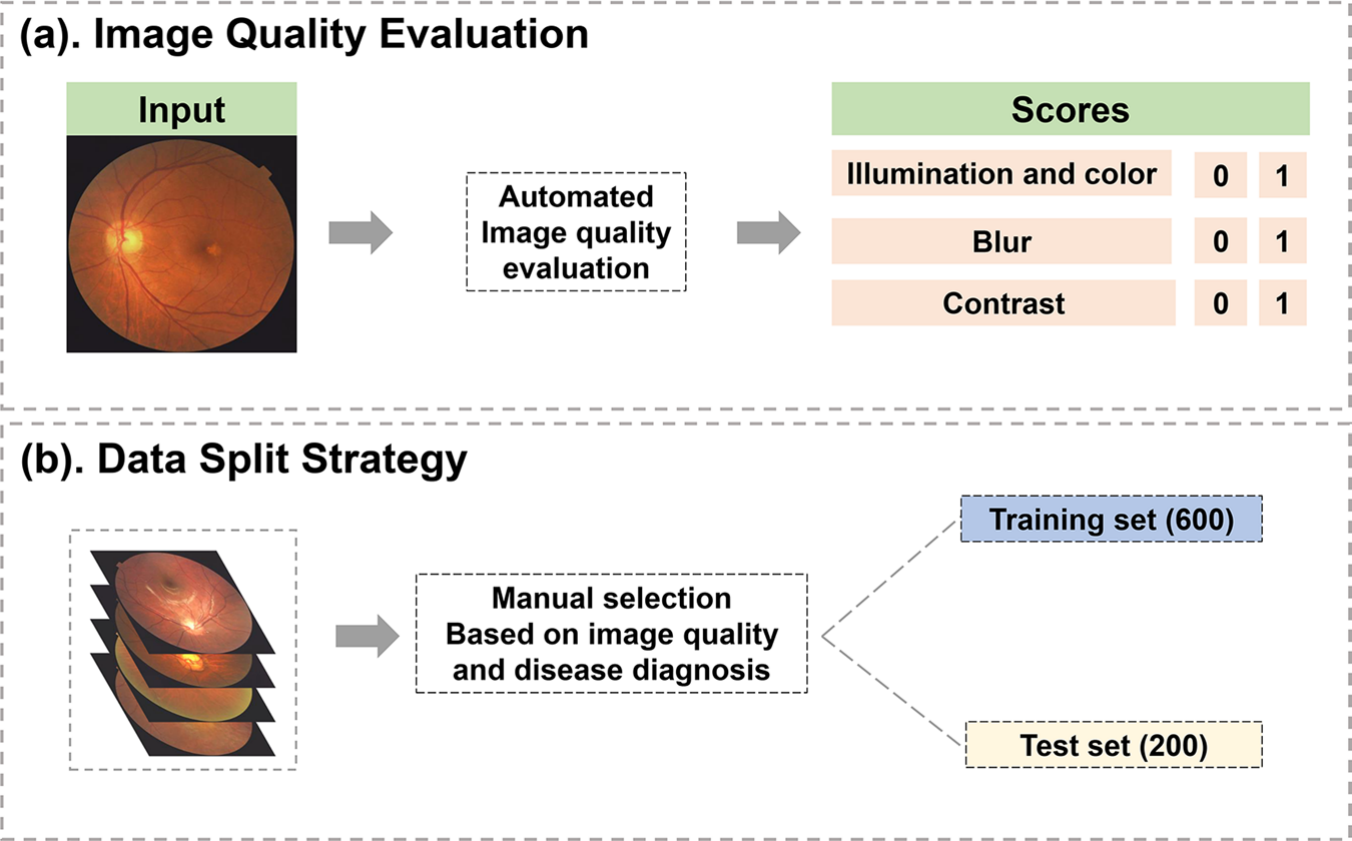
Image quality assessment and data split. (**a**) Illustration of image quality evaluation process. (**b**) Data split strategy.

Considering that the color fundus image quality was substantially influenced by various factors and to make the dataset usable for different research purposes, each image of the FIVES dataset was evaluated on three image quality aspects: illumination and color distortion, blur, and low contrast distortion. The assessment procedure was realized by previously published automatic algorithms^28^. For each aspect, a score, either 0 or 1, was given to represent the quality (Fig. 4a). The image quality gradation grants researchers the opportunity to investigate the robustness of the proposed algorithm.

In AI-based image processing, there should be at least 2 subsets, which are the training set used for learning and the test set used for testing. There should be no intersection between the 2 sets. To fully test an algorithm’s capacity, the characteristics should be distributed equally between these sets. Therefore, we recommend the data split strategy based on the aforementioned image quality score. This process was realized by manual selection (Fig. 4b).

## Data Records

The FIVES dataset has been uploaded to Figshare in the form of a zipped file^29^. The unzipped file folder contains the original fundus photographs, annotation ground truth images and quality assessment scores. The unzipped file is organized into 2 folders and 1 Microsoft office Excel list, named “train”, “test” and “Quality Assessment.xlsx”, respectively. The folders contain the images that are recommended for training and testing. The images in these 2 folders are stored, named and arranged in the same way. In the “train” folder, there are 2 subfolders named “Original” and “Ground Truth” that contain original fundus images and corresponding ground truth images, respectively. Images are named “n_A/D/G/N.png”, where “n” means the number of images and “A”, “D”, “G”, and “N” stand for “AMD”, “DR”, “Glaucoma” and “Normal”, respectively. In the “Ground Truth” folder, images are named according to the same rule, where a specific image in this folder is the ground truth of the image with the same name in the “Original”. “n” is 600 in the training set and 200 in the test set. It should be noted that the current data split strategy is recommended by our team and can be altered for different research purposes. In the file “Quality Assessment.xlsx”, there are 2 sheets titled “Train” and “Test”, with 5 columns in each sheet. The first column represents the disease diagnosis. The second column contains the number of specific images. The subsequent columns represent the *illumination and color distortion*, *blur*, and *low contrast* scores. The image assessment score is either 1 or 0, with 1 indicating good quality in this specific aspect and 0 indicating poor quality.

## Technical Validation

### Dataset characteristics

There are 800 fundus images and their corresponding ground truth images in the FIVES dataset. These pictures are from 573 subjects. The mean age of the subjects was 48 years, with a standard deviation of 19.87 years. There were 469 images from female subjects and 331 from males. All the subjects were Asian. The number and proportion of annotated vessel pixels are summarized in Table 2. The mean image quality scores are summarized in Table 3. Most images were of high quality in terms of the 3 aspects. It is worth noting that images from glaucoma patients tend to have fewer annotated pixels and lower illumination and color distortion scores. We suspect this is because most glaucoma patients are older and may have more opacity in their refractive media, causing insufficient illumination, which would then influence the annotation ratio. The inclusion of some low-quality images can help reflect clinical reality and test the algorithms’ robustness.

**Table 2:** Amount and proportion of annotated pixels in FIVES dataset.

	AMD	DR	Glaucoma	Normal	Total
Amount (Mean)	313671	287402	270192	371171	299561
Proportion (Mean%)	7.48	6.85	6.44	8.85	7.14

**Table 3:** Summarization of image quality assessment scores.

	Illumination and colour distortion (n,%)	Blur (n,%)	Low contrast (n,%)
AMD	187 (83.5%)	178 (89.0%)	200 (100.0%)
DR	172 (86.0%)	154 (77.0%)	198 (99.0%)
Glaucoma	107 (53.5%)	140 (70.0%)	167 (83.5%)
Normal	178 (89.0%)	197 (98.5%)	200 (100.0%)
Overall	644 (78.0%)	669 (83.5%)	765 (95.5%)

### Intra- and inter-annotator consistency

There are some automatic retinal vessel segmentation algorithms that can be used to segment vessels automatically. However, to the best of our knowledge, the best performance of the proposed algorithms yielded a Dice coefficient of approximately 0.82, the Dice coefficient being a value that reflects the accuracy of segmentation^19^. For this specific task, the accuracy is not satisfiable enough to generate a reliable dataset, which is why we opted for manual annotation. In this work, every image was annotated by 2 junior annotators and refined by 1 senior annotator. Therefore, three kinds of consistencies should be investigated: intra-annotator consistency of the same annotator at different times, inter-annotator consistency of the same-level annotators and inter-annotator consistency of different-level annotators. Corresponding experiments were conducted to investigate these kinds of error.

For intra-annotator consistency of the same annotator at different times, 40 images of the whole dataset were selected and extracted to form an example set based on the disease diagnosis and image quality. The example set consisted of 10 images of each diagnosis category and 5–8 low-quality images of each image quality assessment aspect. Five annotators from the annotation group, 1 senior and 4 junior annotators, were asked to annotate the 40 images 2 times in one month, with at least a 1-week gap between each annotation. The Dice coefficient was calculated between the 1st and 2nd annotations. Mean Dice was computed for all 40 images and 4 annotators. The mean Dice was 0.9679 (0.9602–0.9810), which means the annotators’ labeling was stable and the intra-annotator error was small.

For inter-annotator consistency of the same-level annotators, the error was evaluated using Dice between annotations generated by 2 annotators. The original annotation results without refinement were used to investigate this error. The mean Dice was 0.9241 (0.8792–0.9823), which means that the annotation results were close between junior annotators.

For inter-annotator consistency of different-level annotators, the 1st annotations were used for the analysis in this question. The Dice coefficient was calculated between 1 senior annotator’s vessel mask and 4 junior annotators’ annotations. Mean dice was computed. The mean Dice of the annotators was 0.9608 (0.9564–0.9676), which means that the disagreement among annotators of different levels was small.

Through the analysis of original annotations and the example set, we can conclude that annotations were stable and consistent between one annotator at different times and different annotators, which lays the groundwork for accurate annotation and repeatable retinal vessel segmentation.

## Usage Notes

The whole dataset can be downloaded from the link mentioned above. It is worth noting that the data split strategy was made considering the image quality and disease diagnosis. We recommend that users follow this strategy to make the dataset balanced. However, they can always split the strategy according to their study design. For researchers who use traditional algorithms rather than artificial intelligence, the data split is not applicable. Users should cite this paper in their research output and acknowledge the contribution of this dataset in their study.

## Data Availability

No novel code used in the construction of FIVES dataset.
